# Noble Metal Nanoparticle-Based Photothermal Therapy: Development and Application in Effective Cancer Therapy

**DOI:** 10.3390/ijms25115632

**Published:** 2024-05-22

**Authors:** Shujie Yu, Guoyu Xia, Nan Yang, Longlong Yuan, Jianmin Li, Qingluo Wang, Dingyang Li, Lijun Ding, Zhongxiong Fan, Jinyao Li

**Affiliations:** 1 School of Pharmaceutical Sciences and Institute of Materia Medica, Xinjiang University, Urumqi 830017, China; 2 College of Life Science and Technology, Xinjiang University, Urumqi 830000, China

**Keywords:** noble metal nanomaterials, photothermal therapy, tumor therapy, combination therapy

## Abstract

Photothermal therapy (PTT) is a promising cancer therapy modality with significant advantages such as precise targeting, convenient drug delivery, better efficacy, and minimal adverse effects. Photothermal therapy effectively absorbs the photothermal transducers in the near-infrared region (NIR), which induces the photothermal effect to work. Although PTT has a better role in tumor therapy, it also suffers from low photothermal conversion efficiency, biosafety, and incomplete tumor elimination. Therefore, the use of nanomaterials themselves as photosensitizers, the targeted modification of nanomaterials to improve targeting efficiency, or the combined use of nanomaterials with other therapies can improve the therapeutic effects and reduce side effects. Notably, noble metal nanomaterials have attracted much attention in PTT because they have strong surface plasmon resonance and an effective absorbance light at specific near-infrared wavelengths. Therefore, they can be used as excellent photosensitizers to mediate photothermal conversion and improve its efficiency. This paper provides a comprehensive review of the key role played by noble metal nanomaterials in tumor photothermal therapy. It also describes the major challenges encountered during the implementation of photothermal therapy.

## 1. Introduction

Cancer persists as a formidable global health challenge, acknowledged as one of the most lethal diseases [1]. The imperative for enhanced therapeutic interventions has become increasingly evident as the quality of life for patients remains compromised by the adverse effects of existing therapies. It is estimated that in 2022, there will be nearly 20 million new cancer cases and 9.7 million deaths worldwide (including NMSC) [2].

Cancer, with its multifaceted pathology, is mainly treated by traditional modalities such as chemotherapy, radiotherapy, and surgery, but is often accompanied by debilitating side effects [3]. Therefore, novel therapies such as photodynamic therapy (PDT), sonodynamic therapy (SDT), and photothermal therapy (PTT) have been explored in the search for more effective and less harmful therapies [4,5]. Among them, PTT is a promising modality that utilizes a photothermal transforming agent (PTA) to convert light energy into heat energy, thereby inducing localized thermal therapy to ablate tumor cells while minimizing collateral damage to healthy tissues [6]. However, the efficacy of PTT critically depends on the photothermal-converting ability of the photothermal agents, especially the nanoscale variants, which are adept at generating sufficient thermal energy upon light irradiation [7,8]. Therefore, the selection of effective photothermal agents is the key to promoting the efficacy of PTT. In recent years, noble metal nanomaterials have become the frontrunners in this field and won widespread attention due to their huge specific surface area and unique optical, electrical, and catalytic properties [9,10,11]. The application of noble metals in PTT is attributed to their pronounced surface plasmon resonance, which contributes to the effective absorption of specific wavelengths of light in the near-infrared (NIR) spectrum and their subsequent conversion into thermal energy, thereby generating localized thermal therapies conducive to tumor ablation [12,13]. In conclusion, the flourishing development of noble metal nanoparticles in the biomedical field not only circumvents the limitations of traditional tumor therapies, but also heralds the arrival of a new, safe, and minimally invasive mode of cancer therapy.

**Figure 1:**
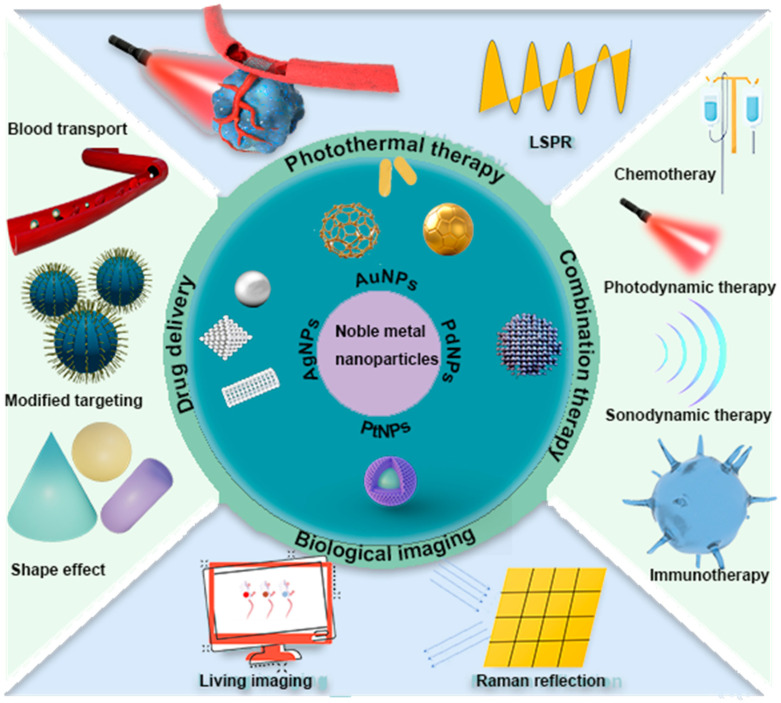
Schematic representation of noble metal nanomaterials for use in cancer photothermal therapy.

Unlike other reviews summarizing the use of noble metals in photothermal therapy for cancer therapy [14,15,16], in this review article, to better understand the use of PTT in tumor therapy, we focus on summarizing the mechanistic studies of noble metals in PTT applications and systematically describe the use of noble metal nanoparticles (including gold, silver, platinum, and palladium) in the field of cancer therapy, presenting combined strategies involving PTT and other therapies, e.g., the application of gold and silver nanoparticles in photothermal therapy (PTT) for cancer therapy is described in detail. This is followed by a comprehensive overview of recent advances in noble metal nanomaterials for cancer therapy, including their role in drug delivery, bioimaging, and combination therapy (Table 1). In addition, we discuss the importance and potential of noble metal nanomaterials-mediated PTT and further suggest future directions for PTT to achieve clinical anti-cancer effects (Figure 1).

## 2. Mechanistic Study of Noble Metal Nanoparticles for Photothermal Therapy in Cancer Therapy

With the continuous development of nanotechnology, metal nanoparticles with diverse functions and rich biological effects have received extensive attention [17]. Metal nanoparticles have many advantages such as controllable size and morphology, excellent optical properties, and easy preparation. Most importantly, metal nanoparticles have both enhanced diagnostic and therapeutic effects and can be used as diagnostic and therapeutic agents, which have important applications in the biomedical field. [18].

Photothermal therapy is a method that uses near-infrared (NIR) light to irradiate a photothermal agent to increase tissue temperature, causing local tissue necrosis through protein denaturation, cell membrane rupture, and DNA damage to achieve tumor killing [19]. The process utilizes the photothermal effect, in which the photothermal agent, after absorbing photons generated by laser irradiation, increases in energy and transforms from the basal unilinear state to an excited unilinear state, which is unstable and then returns to the basal state through non-radiative vibrational relaxation (collision of the photothermal molecules with the molecules of the surrounding material to dissipate the energy), resulting in the effect of warming up the temperature [20]. Among these, the extraordinary effects produced by noble metals as photothermal agents for this therapy have attracted significant attention. Noble metal nanoparticles have powerful surface plasmon resonance (SPR) properties, which means they can absorb specific wavelengths of light efficiently, especially in the near-infrared region (NIR). NIR light is highly penetrating and penetrates deep into tissues, reducing damage to surrounding normal tissues. When a noble metal nanoparticle absorbs light energy, it converts the light energy into heat energy through a non-radiative relaxation process. This process leads to a significant increase in the local temperature around the particle [21]. The local high temperatures generated by noble metal nanoparticles can directly disrupt the structure and function of tumor cells, causing protein denaturation, cell membrane damage, and even destruction of cellular organelles, such as mitochondrial dysfunction thus leading to an insufficient energy supply. Of course, the local thermal effect can also lead to cell death in a variety of ways, including necrosis (death of cells that are damaged to the point that they are unable to sustain life activities) and apoptosis (programmed cell death, controlled by intracellular signaling pathways) [22]. Apart from these, high temperatures may also trigger cellular autophagy and inflammatory responses. In addition to directly killing tumor cells, local thermal effects may activate the immune system and promote tumor recognition and attack by immune cells. Released tumor antigens can stimulate the immune system to respond more strongly to tumors, even to untreated metastatic tumor cells. Of course, the local thermal effect can also damage tumor blood vessels and weaken their blood supply, further enhancing the killing effect on tumor cells [23].

As plasmonic excitation element metals, the LSPR of noble metal nanoparticles, such as Au and Ag, are sensitive to many factors, such as their size, shape, composition, environment, and interaction with neighboring nanoparticles [24]. Therefore, we can not only inhibit the light scattering at the LSPR or increase the light absorption of PNPs, which can improve the photothermal conversion, but also tune the size and shape (morphology) of the PNPs as well as the composition mainly to improve the photothermal conversion. The researchers synthesized three common gold nanostructures, namely gold nanospheres (AuNSs), gold nanorods (AuNRs), and gold nanostars (AuNSTs), by the same mPEG-SH surface modification. The results show that all AuNPs can convert 808 nm near-infrared (NIR) laser light energy into thermal energy through the localized surface plasmon resonance effect, with the AuNSTs exhibiting the highest photothermal conversion efficiency [25]. Based on this, whether the crystallinity of the PNPs (plasmonic nanostructures) affects the photothermal conversion efficiency has also been investigated in recent years. In this study, the researchers developed a defect-damped harmonic oscillator model. Model calculations show that defect-induced damping can effectively reduce the light scattering of PNPs and significantly improve their PCE, especially for PNPs with sufficiently large sizes (Au and Ag greater than ~100 nm), and they have found that defect-induced damping significantly improves their light absorption and photothermal performances [26]. Certainly, the shapes of the silver nanoparticles have a significant effect on the photothermal conversion efficiencies, such as quasi-spherical silver nanoparticles, silver nanorods, silver nanocubes, Ag–Rh core-framework nanocubes, and silver prismatic nanocubes [27]. Among all the shapes of silver materials, silver nanoprisms have great potential in photothermal therapy (PTT) due to their strong surface plasmon resonance bands in the near-infrared region [28].

In summary, modern medicine utilizes noble metal nanoparticles (AuNPs) of specific shapes and sizes to provide a relatively mild alternative to cancer diagnosis and therapy by absorbing near-infrared (NIR) light and generating a plasmonic resonance effect for two main purposes, namely enhancing tumor detection and generating localized heat at the tumor site for thermal ablation. Noble metal nanoparticles, which act as light absorbers, can be injected into the tumor area, and then produce a thermal effect on the tumor under light excitation. This thermal effect can raise the temperature of the tumor region to a sufficiently high level in the time required to achieve tumor destruction [29].

## 3. Noble Metal Nanomaterials in Cancer Therapy

### 3.1. Gold Nanoparticles

#### 3.1.1. Photothermal Therapy

Photothermal therapy (PTT) holds significant promise in tumor therapy owing to its distinctive advantages, including high specificity and minimal invasiveness [30,31]. Gold nanomaterials, leveraging their surface plasmonic properties, serve as efficacious photothermal converters, thus enhancing the photothermal conversion efficiency of PTT [32]. Consequently, gold nanomaterials have emerged as a focal point of scientific inquiry in this domain.

**Figure 2:**
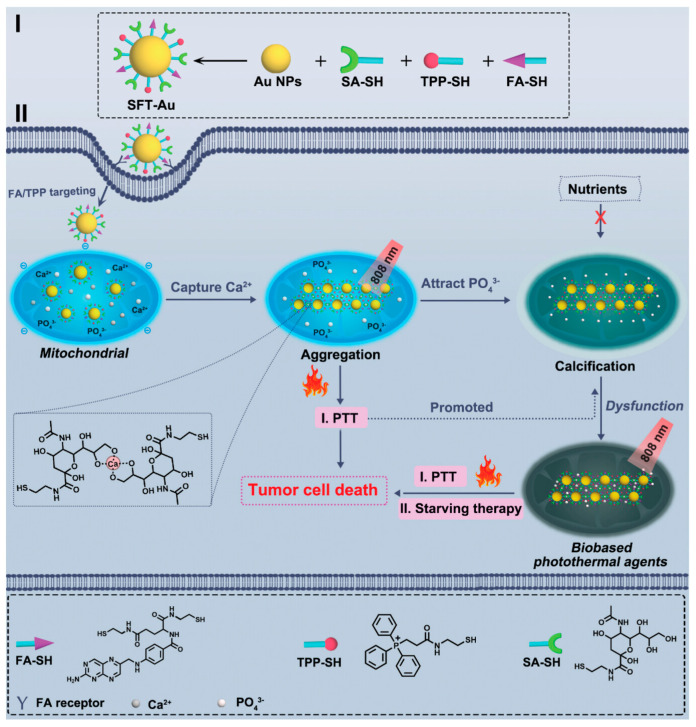
Schematic illustration of synthesis (**I**) and therapeutic mechanism (**II**) of SFT-Au nanoparticles. The nanoparticles were able to utilize and manipulate the over-expressed calcium in the mitochondria of tumor cells for the simultaneous inhibition of malignant tumors via calcium-dependent photothermal therapy and mitochondrial calcification-mediated starving therapy [33]. Copyright 2023, Wiley-VCH GmbH.

Due to the inherent biosafety profile of starvation therapy for inducing tumor calcification, researchers have increasingly focused on this therapeutic modality in recent years. However, the efficacy of this approach is hindered by the limited availability of calcium ions in or around tumor tissues, leading to a slow and uncontrollable physiological calcification process. This challenge necessitates innovative strategies to enhance the effectiveness of starvation therapy. Recently, a group of researchers [33] developed a novel approach by synthesizing gold nanoparticles (designated as SFT-Au) functionalized with salivary acid (SA, a calcium chelator), folic acid (FA, serving as a tumor-targeting moiety), and triphenylphosphine (TPP, facilitating mitochondrial targeting). Leveraging the abundance of mitochondria within the tumor cells and capitalizing on the light collection and photothermal properties inherent to SFT-Au, this multifunctional nanoplatform aimed to achieve precise calcification of tumor mitochondria, thereby enhancing the efficacy of starvation therapy. Evaluation of this nanoplatform in photothermal therapy (PTT) revealed that calcium chelation induced nanoparticle aggregation, resulting in a significant enhancement of absorption in the long wavelength region (Figure 2). This phenomenon can be attributed to the size-dependent absorption characteristics of gold nanoparticles, thereby facilitating calcium-dependent photothermal conversion upon exposure to 808 nm radiation. Importantly, this calcium-dependent photothermal conversion exhibited sustained high efficiency even after multiple cycles, underscoring the remarkable stability of SFT-Au aggregates under near-infrared (NIR) radiation and elevated temperatures and thus holding promise for sustained antitumor therapy.

Due to the involvement of HER2 and HER3 oncogenes in the pathogenesis and progression of specific invasive breast cancers, the overexpression of these genes presents a challenge in achieving therapeutic efficacy against such malignancies. Particularly, HER3 overexpression contributes to resistance mechanisms against conventional antitumor agents. To address these hurdles, Eva Villar-Alvarez et al. [34] devised a multifunctional, biocompatible nanoplatform integrating diagnostic and therapeutic modalities. This platform comprises branched gold nanoshells loaded with doxorubicin, conjugated with the near-infrared (NIR) fluorescent dye indocyanine green, and further functionalized with small interfering RNA (siRNA) targeting HER3, along with the HER2-specific antibody trastuzumab. This design enables a synergistic therapeutic approach, combining chemotherapy, photothermal therapy, RNA interference, and immunomodulation. In vivo experiments conducted in a hormonal mouse model demonstrated a notable reduction in tumor volume following administration of the hybrid nanocarriers, coupled with subsequent near-infrared light exposure. These findings underscore the promising therapeutic potential of such integrated nanoplatforms for combating resistant breast cancer.

#### 3.1.2. Combined Photothermal and Immunotherapy Therapy

Nanomaterial-mediated photothermal therapy (PTT) holds promise for the therapy of localized tumors [35]; however, its efficacy in addressing tumor metastasis and recurrence is constrained. Combination therapy offers a strategy to enhance therapeutic outcomes, leveraging synergistic effects where the combined effect exceeds the sum of individual therapies [36].

**Figure 3:**
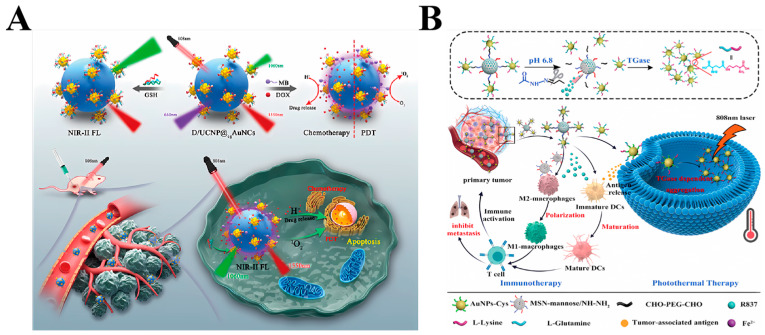
(**A**) Schematic illustration of the D/UCNP@cgAuNCs nanoassemblies for tumor microenvironment-enhanced ratiometric NIR-II fluorescence imaging and chemo-/photodynamic combination therapy [43]. Copyright 2023, American Chemical Society; (**B**) Schematic representation of the therapeutic processes of pH–enzyme–NIR multi-responsive immune-adjuvant nanoparticles (R837@MSNmannose-AuNPs-Glu/Lys, RMmAGL), which combines tumor-specific photothermal therapy and photothermal-assisted immunotherapy for malignant tumor therapy. GNP-VGB3 recognizes VEGFR1 and VEGFR2 and suppresses their VEGF-induced phosphorylation in endothelial cells [38]. Copyright 2023, Wiley-VCH GmbH.

In recent years, PTT has emerged as a prominent modality in cancer therapy, with nanomaterial-based photoimmunotherapy presenting distinct advantages. This approach facilitates the release of tumor-associated and tumor-specific antigens, thereby promoting synergistic immunotherapeutic responses. Despite the advancements in immunotherapy leading to improved survival rates among cancer patients, its clinical benefits are constrained in the context of ‘cold tumors’ characterized by a lack of infiltrating T cells. Xiao et al. [37] devised a tumor-targeting nanosystem named AuNC@SiO_2_@HA, aiming to modulate the immune microenvironment in murine melanoma exhibiting an immunologically ‘cold’ state, thereby eliciting synergistic effects with an immune checkpoint blockade (ICB). To evaluate the therapeutic potential of AuNC@SiO_2_@HA, a subcutaneous transplantation tumor model was established in immunocompetent SMM102 mice. Subsequently, different therapeutic regimens including saline, anti-PD-1 alone, AuNC@SiO_2_@HA combined with laser irradiation, and a combination of anti-PD-1 with AuNC@SiO_2_@HA plus laser irradiation were administered. Tumor growth progression was meticulously monitored throughout the experimental duration. After the completion of therapy on day 18, tumor volume and weight were measured post dissection. The outcomes revealed that anti-PD-1 monotherapy exhibited limited efficacy against tumors, whereas AuNC@SiO_2_@HA demonstrated remarkable therapeutic effectiveness against tumors when coupled with laser irradiation. Tao Liu et al. [38] developed pH–enzyme–NIR multi-responsive immunoadjuvant nanoparticles (RMmAGL) tailored for tumor-specific photothermal therapy and photothermal-assisted immune modulation (Figure 3B). Within tumor microenvironments, the acidic conditions triggered the dissociation of AuNPs-Glu/Lys from RMmAGL, facilitating the release of the TLR7 agonist R837. Upon internalization by tumor cells, liberated AuNPs-Glu/Lys aggregated, facilitated by the catalytic activity of TGase which is typically overexpressed in tumor cells. This aggregation enabled tumor-specific photothermal therapy upon NIR irradiation. Importantly, this process not only induces damage to the primary tumor but also prompts the generation of tumor-associated antigens in situ. The tumor-associated antigens facilitate the binding to R837, effectively stimulating the maturation of dendritic cells (DCs) through a mechanism akin to vaccination. This activation subsequently triggers antitumor T cells, thereby promoting immunotherapy. Moreover, the residual MSN mannose present in tumor tissues induces the polarization of tumor-associated macrophages from an M2-type to an M1-type phenotype. This polarization serves to remodel the immunosuppressive tumor microenvironment into an antitumor milieu, thereby further augmenting the efficacy of immunotherapy. HyeMi Kim et al. integrated adoptive cell therapy (ACT) with photothermal therapy (PTT) by incorporating AuNPs into tumor-reactive T cells, which were then administered intravenously [22]. The AuNP-loaded T cells migrated to the tumor tissues and initiated the elimination of tumor cells. However, over time, these T cells gradually lost control over the tumor cells, resulting in tumor regrowth. Leveraging the remarkable tumor-homing capabilities of T cells, a portion of the AuNPs were transported to the tumor tissue. Subsequently, upon the loss of T cell efficacy against the tumor cells within the tumor microenvironment, photothermal therapy (PTT) was employed to further eradicate residual tumor cells. Compared to ACT or PTT monotherapy, the combination of immunophotothermal therapy significantly attenuated tumor growth and improved overall survival. 

#### 3.1.3. Other Combined Therapies

Photothermal therapy (PTT) has garnered significant attention in cancer therapies. However, a notable challenge arises from the upregulation of heat shock proteins (HSPs) in tumor cells following heating, which can counteract the cellular damage induced by elevated temperatures. This phenomenon poses a substantial limitation to the efficacy of PTT as a standalone therapeutic modality. To enhance therapeutic outcomes, it is imperative to explore synergistic approaches wherein PTT is combined with other modalities such as immunotherapy, chemotherapy, radiotherapy, and other established cancer therapies. This integrated approach holds promise for achieving enhanced therapeutic efficacy and ultimately improving patient survival rates [39]. Gold nanorods have garnered significant attention in the field of tumor thermochemotherapy. Specifically, gold nanorods (AuNRs) exhibit tunable longitudinal absorption spectra within the near-infrared laser (NIR) region, thereby presenting immense potential for cancer photothermal therapy [40]. Gold nanorods (AuNRs) have encountered challenges in achieving efficient application in vivo for photothermal therapy due to limited thermal availability. To overcome this obstacle, Zhao et al. [41] devised AuNR-based nanocomplexes (NCs) with enhanced responsiveness, leveraging the synergistic effects of photothermal therapy and chemoembolization. The AuNR core and doxorubicin (DOX) were encapsulated within N-(2-hydroxypropyl) methacrylamide (HPMA)-co-N-(1-vinyl-2-pyrrolidone) (NIPAM) copolymer nanoparticles (NPs) via electrostatic and hydrophobic interactions, respectively. Upon intravenous administration, NIR irradiation-induced temperature elevation prompted a phase transition of NIPAM, facilitating NC aggregation and the subsequent blockade of tumor vasculature. This process facilitated the release and transvascular transport of DOX, leading to its accumulation within the tumor. Consequently, the combined action of DOX and AuNRs resulted in localized antitumor efficacy while minimizing adverse effects on non-tumor tissues.

Certainly, photodynamic therapy (PDT) stands out as a highly effective localized therapy for tumors, relying on reactive oxygen species (ROS) to induce cell death via the utilization of the singlet oxygen generated by excited-state photosensitizers under suitable light sources. However, despite its efficacy, PDT encounters challenges stemming from factors such as low oxygen levels, the short half-life of ROS, and the limited availability of photosensitizers delivered via the intravenous (IV) route, as well as the constrained accumulation of photosensitizers at the tumor site. These limitations hinder PDT’s broader application in tumor therapy. Addressing these issues, Xiaodong Ma et al. [42] successfully achieved the integration of photothermal therapy (PTT) and PDT using a singular nanocarrier strategy. They employed Au@MSN nanoparticles as carriers for the intracellular delivery of the photosensitizer tetra(4-hydroxyphenyl)porphyrin (THPP). The resulting Au@MSN-Ter/THPP@CM nanoparticles exhibited remarkable photothermal conversion capabilities and demonstrated efficient uptake by ovarian cancer cells. Both the Au@MSN-Ter/THPP@CM nanoparticles and Au@MSN-Ter/THPP@CM@GelMA/CAT mimetic nano@microgels exhibited significant inhibition of cell proliferation.

Tumor microenvironment-mediated ratiometric near-infrared two-region (NIR-II) fluorescence imaging and photodynamic therapy play pivotal roles in enabling accurate diagnosis and effective therapy of deep-seated tumors. However, integrating these functionalities within a single nanoparticle remains a considerable challenge. Shengqiang Hu et al. [43] have addressed this issue by developing novel single-excitation triple-emission down/up-conversion nanoassemblies (Figure 3A). These assemblies enable simultaneous GSH-enhanced ratiometric NIR-II fluorescence imaging and chemo/photodynamic combination therapy for tumors.

#### 3.1.4. Biological Imaging

The detection of biomolecules holds paramount significance in fundamental molecular research, diagnostics [44,45], drug screening, and various biomedical applications. Raman spectroscopy, an analytical technique, relies on the scattering of photons by molecules within a sample to measure their vibrational and rotational modes [46,47]. This method is not only facile to execute but also rapid and non-destructive, circumventing interference from aqueous solutions. Importantly, Raman spectroscopy provides highly accurate information regarding the molecular composition and structure of the target molecules [48].

However, the extensive clinical utility of Surface-Enhanced Raman Scattering (SERS) detection is hindered by the absence of a standardized method for concurrently detecting enhanced Raman signals emanating from diverse biomolecules. In response to this challenge, researchers have devised a universal SERS detection platform leveraging gold nanoparticles (AuNPs) to analyze a wide array of biomolecules. Utilizing a two-step enhancement strategy, distinct signatures of various biomolecules such as DNA, RNA, amino acids, peptides, proteins, viruses, bacteria, and lipid molecules can be directly discerned through the measurement of SERS signals, obviating the need for labeling [49]. Gold nanostructures facilitate light concentration at the nanoscale by the resonance excitation of their free electrons, a phenomenon known as surface plasmonics. In Surface-Enhanced Raman Scattering (SERS), an intensified electromagnetic field amplifies Raman-scattered light emitted by proximal molecules. Gold nanostructures function as antennas, concentrating light onto molecules and enhancing the Raman-scattered signal to enable the recording of individual molecules’ vibrational spectra [50]. The strength of the field directly impacts the resolution and sensitivity of analytical techniques like Surface-Enhanced Raman Scattering (SERS). Consequently, “field focusing” has emerged as a crucial research focus, with strategies such as creating dense near-field spots or hot spots employed to enhance near-field focusing effectiveness. Building on this concept, researchers have successfully synthesized gold nanohalos of varying sizes using multiple stepwise synthesis pathways. They demonstrated effective near-field focusing for different gap distances between the inner and outer nanohalos through single-particle SERS measurements [51].

#### 3.1.5. Drug Delivery

Gold nanoparticles exhibit significant potential in biomedical applications, particularly in drug delivery and cancer therapy, owing to their distinctive physical and chemical characteristics. These nanoparticles can be tailored in various sizes and shapes, influencing their biodistribution and cellular uptake dynamics [25]. Consequently, gold nanoparticles can accumulate within tumor tissues through passive targeting mechanisms, leveraging the enhanced permeability and retention (EPR) effect, as well as through active targeting strategies, which involve surface modifications with molecules designed to recognize specific targets. The researchers investigated the potential of nuclear-targeted gold nanoparticles for radiosensitization in pancreatic cancer. They achieved this by utilizing nuclear localization sequence (NLS) peptides to target gold nanospheres, thereby enhancing the accumulation of these particles within the cell nucleus. The experimental findings indicate that the targeted delivery of gold nanoparticles to the nucleus results in amplified radiosensitization through the augmentation of DNA double-strand break formation [52]. In a related context, researchers have explored the use of a VEGFA/VEGFB antagonist peptide (VGB3) coupled with gold nanoparticles to improve efficacy and extend the therapeutic duration. VGB3 is known for its ability to recognize and neutralize VEGFR1 and VEGFR2 on both endothelial and tumor cells [53]. When bound to gold nanoparticles (GNP-VGB3), it effectively identifies VEGFR1 and VEGFR2 in endothelial cells, leading to the inhibition of VEGF-induced phosphorylation of these receptors. Importantly, VGB3 would maintain its capacity to recognize VEGFR1 and VEGFR2 even after binding to gold nanoparticles. Furthermore, therapy with GNP-VGB39 resulted in the inhibition of VEGF-induced phosphorylation of both VEGFR2 and VEGFR1. These findings strongly suggest that GNP-VGB3 effectively impedes the VEGF-induced activation (phosphorylation) of VEGFR1 and VEGFR2. Moreover, the utilization of gold nanoparticles in photothermal therapy (PTT) offers notable targeting capabilities. Gold nanoparticles possess the ability to generate heat upon exposure to near-infrared light (NIR), enabling their application in localized heating-based cancer therapy. This targeted heating effect can be directed toward tumor sites where gold nanoparticles accumulate [54]. In recent studies, researchers have explored the combined use of gold nanoshell (NS) technology with photothermal therapy (PTT) and liposomal doxorubicin to enhance the prognosis of mouse models with colorectal cancer. The results demonstrated that the combination of PTT with liposomal doxorubicin led to a deceleration in tumor growth rate and an improvement in the survival rate of the mice [55].

The precise targeting capabilities inherent in gold nanoparticles, achieved through meticulous design and customization, render them highly promising entities in the realm of cancer therapy. These nanoparticles exhibit the potential to enhance the efficiency and precision of drug delivery while mitigating the adverse effects on healthy tissues. However, it is imperative to note that further research and optimization are essential to address concerns about the safety, stability, and biodistribution of gold nanoparticles in clinical applications.

### 3.2. Silver Nanoparticles

#### 3.2.1. Photothermal Therapy

As one of the extensively employed nanoparticles in both biomedical and industrial realms, silver nanoparticles exhibit a diverse array of effects, including antibacterial [56], anti-inflammatory [57], and antitumor properties [58]. Research indicates that silver nanoparticles manifest low toxicity in their nanoparticulate form. Conversely, Ag+, generated under oxidizing conditions, demonstrates heightened cytotoxicity against various cancer cell lines through the induction of oxidative stress, mitochondrial damage, and autophagy. The remarkable physicochemical properties inherent in silver nanoparticles render them suitable for applications in Surface-Enhanced Raman spectroscopy (SERS) and metal-enhanced fluorescence [18,59]. Furthermore, silver nanoparticles demonstrate immunomodulatory and radiosensitizing effects [58,60].

**Figure 4:**
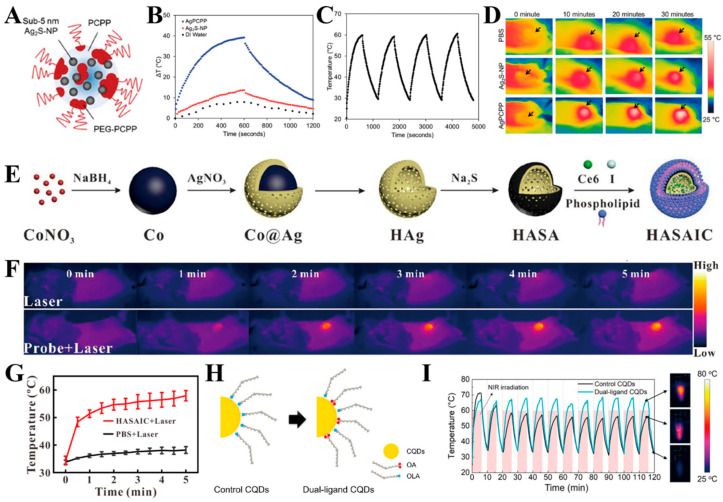
(**A**) Schematic illustration of AgPCPP nanoparticles; (**B**) Heating and cooling curves of AgPCPP nanoparticles, free Ag_2_S-NP and DI water under 10 min of irradiation (808 nm, 2 W cm^−2^); (**C**) Photostability of AgPCPP under four cycles of irradiation/cooling for a total duration of 20 min (808 nm, 2 W cm^−2^); (**D**) Infrared thermography of tumors treated with saline, Ag_2_S-NP, and AgPCPP nanoparticles during 30 min of laser irradiation [61]. Copyright 2023, Wiley-VCH GmbH; (**E**) Schematic diagram of the synthesis of the HASAIC probe; (**F**) Temperature change at tumor site under laser irradiation after injection of PBS and probe, respectively; (**G**) Temperature changing curve of tumor site under laser irradiation after injection of PBS and probe [62]. Copyright 2021, Elsevier B.V. All rights reserved; (**H**) Schematic illustration of ligand passivation on the Ag_2_S CQD surface; (**I**) On-off cycles of the photothermal effect with 500 nM CQDs under 1.5 W/cm^2^ [64]. Copyright 2024, American Chemical Society.

Silver sulfide nanoparticles (Ag_2_S-NP) hold considerable promise in optics-based biomedical applications, including near-infrared fluorescence (NIRF) imaging, photoacoustic (PA), and photothermal therapy (PTT). Addressing the limitations of conventional silver sulfide nanoparticles, characterized by low NIR light absorbance, stringent preparation conditions, and the use of toxic precursors, [A Biodegradable] and colleagues successfully synthesized Ag_2_S-NP with a size below 5 nm. These nanoparticles were then encapsulated in biodegradable polymer nanoparticles (AgPCPP) (Figure 4A). This innovative approach, employing non-toxic materials and mild preparation conditions, resulted in an increased number of silver sulfide encapsulations within the nanoparticles, thereby enhancing their NIR absorption and subsequently improving optical imaging and PTT effects [61] (Figure 4B–D). In a non-coincidental manner, Zhang et al. orchestrated the synthesis of a hollow Ag_2_S/Ag nanocomposite shell, comprising monolithic Ag and compound Ag_2_S. Following the incorporation of acoustic sensitizers and CT contrast agents, they achieved the pioneering development of multifunctional HASAIC nanoprobes through the envelopment of a thermally supported lipid bilayer (Figure 4E). The monolithic Ag within the probe demonstrated catalytic prowess, facilitating the conversion of H_2_O_2_ into O_2_. This catalytic activity serves to mitigate the anoxic conditions at the tumor site, thereby augmenting the effectiveness of acoustic power therapy. Notably, the incorporation of a hollow Ag_2_S/Ag nanocomposite shell layer serves a dual purpose in that it prevents the dissolution of the pure Ag shell layer during the catalytic generation of O_2_ from H_2_O_2_, consequently avoiding undesirable consequences in the context of photothermal therapy (PTT) (Figure 4F,G) and photoacoustic imaging (PAI) [62].

**Figure 5:**
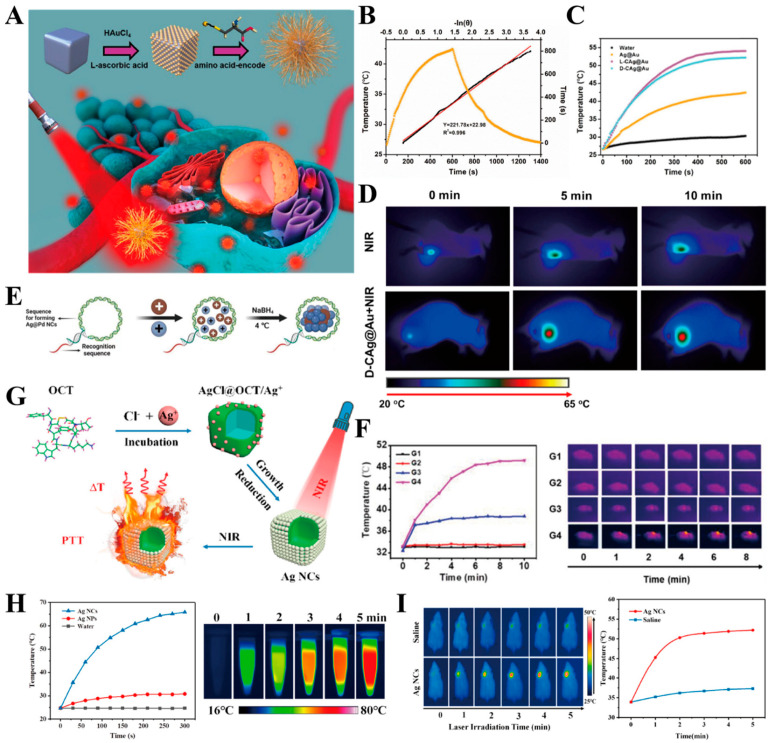
(**A**) Schematic illustration of L/D-CAg@Au nanoparticles. (**B**) Calculation of the photothermal conversion efficiency at 808 nm. The orange curve represents the photothermal effect of the Ag@Au aqueous solution. The black line represents the time constant (τs) for the heat transfer of the system. (**C**) Heating and cooling curves of water, Ag@Au, L-CAg@Au, and D-CAg@Au aqueous dispersion (100 μg mL^−1^) under the 808 nm laser on/off irradiation (1.0 W cm^−2^). (**D**) Infrared thermography of tumor sites exposed to 808 nm laser irradiation at 1.0 W cm^−2^ for 10 min [13]. Copyright 2023, American Chemical Society. (**E**) Schematic Illustration of the Synthesis of AgNCs Using OCT as the Biotemplate and their application. (**F**) Photothermal heating curves of MKN-45 tumors under 1270 nm laser after intravenous injection of DNA-Ag@Pd NCs for 6 h [63]. Copyright 2023, Wiley-VCH GmbH. (**G**) Schematic illustration of the Synthesis of DNA-templated Ag@Pd alloy nanoclusters (DNA-Ag@Pd NCs). (**H**) The temperature elevation profiles of AgNP and AgNC solutions at the same concentration under laser irradiation and the photothermal images of the AgNCs solution at different time intervals irradiated. (**I**) In vivo thermal images of mice injected with saline or the AgNCs under NIR laser irradiation and tumor temperature profiles as the function of laser irradiation time [65]. Copyright 2018, American Chemical Society.

Liu et al. engineered a distinctive black noble metal core–shell nanostructure featuring silver (Ag) nanocubes as the core and amino acid-encoded highly branched gold (Au) nanorods as the shell (L-CAg@Au and D-CAg@Au) (Figure 5A). Both L-CAg@Au and D-CAg@Au showcased superior photothermal conversion properties when compared to the amino-acid-free core–shell structure (Ag@Au) (Figure 5B,C). The antitumor therapeutic efficacy of the synthesized samples underwent a comprehensive evaluation both in vitro and in vivo. Apoptosis analysis, conducted through flow cytometry, revealed that D-CAg@Au serves as a potent photothermal therapeutic agent for antitumor applications by inducing apoptosis under laser irradiation, demonstrating commendable therapeutic effectiveness and biosafety (Figure 5D) [13]. Zhang et al. utilized a cytosine-rich hairpin-like DNA structure as a growth template to fabricate a novel class of noble metal alloy nanoenzymes termed DNA template Ag@Pd alloy nanoclusters (DNA-Ag@PdNCs) (Figure 5E). These nanostructures, characterized by the integration of silver (Ag) and palladium (Pd) within the DNA scaffold, demonstrated remarkable properties. Specifically, under 1270 nm laser irradiation, the DNA-Ag@PdNCs exhibited an impressive photothermal conversion efficiency of 59.32%. Additionally, a synergistic enhancement of peroxide mimicry enzyme activity was observed due to the unique interplay between the Ag and Pd constituents. The presence of a hairpin DNA structure on the surface of the DNA-Ag@PdNCs conferred several advantageous attributes. Firstly, it imparted excellent stability and biocompatibility ex vivo, rendering these nanostructures suitable for biological applications. Moreover, the DNA scaffold contributed to an enhanced tumor site permeability and retention effect, facilitating targeted delivery and accumulation within tumor tissues. Upon intravenous administration, DNA-Ag@PdNCs demonstrated significant promise as a theranostic agent for gastric cancer. Utilizing high-contrast NIR-II photoacoustic imaging guidance, efficient photothermal enhancement was achieved, augmenting the efficacy of nanocatalytic therapy (NCT). Experimental findings corroborated the ability of DNA-Ag@PdNCs to effectively inhibit gastric cancer tumor growth and eradicate tumor cells through the synergistic effects of photothermal therapy (PTT) and NCT (Figure 5F). In summary, DNA-Ag@PdNCs represent a multifunctional platform for tumor diagnosis and therapy, holding great potential as a versatile tool in nano-diagnostic and therapeutic applications [63]. Yoo et al. utilized oleic acid and oleylamine as the co-ligands for surface passivation to achieve the enhanced confinement of the CQD morphology, effectively prevented the CQD fusion, and prepared high monodispersity silver sulfide (Ag_2_S) colloidal quantum dots (CQDs) for tumor diagnosis and therapy (Figure 4H). Experimental results showed that the CQDs that were synthesized using dual ligands exhibited uniform size distribution, showed efficient photothermal effects under near-infrared laser irradiation (Figure 4I), and were able to effectively kill tumor cells [64]. Bian et al. drew inspiration from biomineralization processes to fabricate silver-based peptide-directed mineralized silver nanocages (AgNCs). These AgNCs represent organic–inorganic hybrids synthesized utilizing octreotide (OCT) as a template, with their shells composed of AgNPs (Figure 5G). This hierarchical architecture ensures tight aggregation of the AgNPs, thereby facilitating exceptional plasmonic coupling. Consequently, there is a notable redshift in the resonant excitation wavelength from the visible spectrum (420 nm) to the near-infrared (NIR) region (810 nm). Moreover, the manipulation of the size and morphology of mineralized AgNCs through the modulation of the volume of added silver nitrate (AgNO_3_) enables precise control over the surface plasmon resonance peak of AgNCs within the NIR spectrum. Experimental findings illustrate that AgNCs exhibit a photothermal conversion efficiency of 46.1%, selectively inducing cancer cell death upon NIR irradiation at 808 nm (Figure 5H). These AgNCs demonstrate remarkable antitumor properties and exhibit favorable biocompatibility in the context of photothermal therapy (Figure 5I) [65]. 

#### 3.2.2. Combined Photothermal and Immune Therapy

Tumor immunotherapy represents a crucial therapeutic approach in cancer therapy, leveraging the body’s immune system to combat tumors. It has emerged as a pivotal strategy alongside conventional modalities such as surgery, chemotherapy, radiotherapy, and targeted therapies, offering notable clinical efficacy and advantages [66].

Immunotherapeutic strategies encompass a diverse range of interventions, including therapeutic vaccines, immune checkpoint blockade, bispecific T-cell engagers (BiTEs), and adoptive cell therapy [67]. Among these, immune checkpoints serve as pivotal regulators of T-cell activation [68]. They play a crucial role in maintaining immune homeostasis by preventing excessive activation of the immune system or autoimmune responses through inhibition of T-cell activation. Tumor cells exploit this mechanism to evade immune surveillance. Conversely, immune checkpoint inhibitors, typically comprising small molecule drugs or antibodies, counteract checkpoint-mediated immunosuppression by competitively binding to immune checkpoints. This blockade unleashes T-cell activation, thereby enabling the immune system to mount a robust antitumor response and eliminate tumor cells.

**Figure 6:**
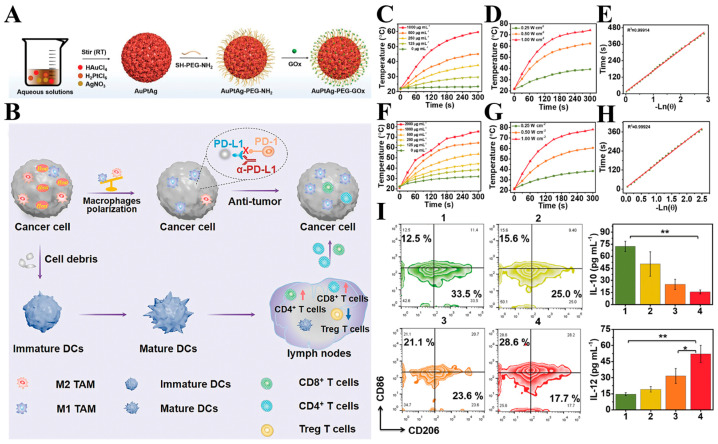
(**A**) Schematic illustration of the formation of AuPtAg-GOx nanozyme. (**B**) The mechanism for tumor immunotherapy induced by starvation therapy-augmented mild photothermal therapy. (**C**,**F**) Temperature changes of AuPtAg at different concentrations under 808 nm and 1064 nm laser irradiation (0.5 W cm^−2^, 5 min). (**D**,**G**) Temperature changes of AuPtAg (1000 μg mL^−1^) with various laser power densities (808 nm laser (**D**) and 1064 nm laser (**G**) for 5 min). (**E**,**H**) Plot of cooling time versus negative natural logarithm of the temperature driving force (808 nm laser (**E**) and 1064 nm laser (**H**)). (**I**) Representative flow cytometer plots of M2-type macrophages (CD206+), and M1-type macrophages (CD86+) in TAMs (F4/80+) and the secretion levels of IL-10, IL-12 in the supernatant after different therapies. Groups: (1) control, (2) AuPtAg-GOx, (3) AuPtAg-PEG + 1064 nm (0.5 W cm^−2^), (4) AuPtAg-GOx + 1064 nm (0.5 W cm^−2^). Statistical significance is assessed by an unpaired Student’s two-sided *t*-test. * *p* < 0.05, ** *p* < 0.01 [71].

In recent years, immune checkpoint blockade therapy has solidified its position as a pivotal strategy in tumor immunotherapy. Monoclonal antibodies targeting checkpoint molecules, including programmed death receptor 1 (PD-1)/programmed death ligand 1 (PD-L1) and cytotoxic T-lymphocyte antigen 4 (CTLA-4), have received FDA approval and demonstrated success in clinical applications [69,70]. The emergence of these therapies marks a significant milestone in the field. The integration of the immune checkpoint blockade therapy with other therapeutic modalities, such as photothermal therapy, has garnered considerable attention. Notably, Wang et al. devised a novel approach involving AuPtAg-GOx nanoenzymes (Figure 6A,B). These nanoenzymes generate controlled heat at the tumor site upon exposure to 1064 nm laser irradiation, exhibiting mild photothermal properties (Figure 6C–H). Importantly, this system demonstrates the capability to alleviate the heat resistance of tumor cells, thereby enhancing the efficacy of the antitumor immune response (Figure 6I). The combination of mild photothermal therapy (PTT) and glucose oxidase (GOx)-mediated starvation therapy synergistically enhances the efficacy of PTT. This synergy is attributed to the improved recruitment of tumor-infiltrating lymphocytes (TILs) and the induction of immunogenic cell death (ICD), consequently transforming the tumor microenvironment from “cold” to “hot”. This addresses the challenge of the limited efficacy of immune checkpoint blockade therapies in treating “cold” tumors. In vivo experiments have demonstrated that the addition of αPD-L1 to the therapy regimen comprising AuPtAg-GOx-mediated mild PTT, starvation therapy, and immunotherapy effectively suppresses both primary and distal tumors [71]. Jin et al. pioneered the development of corn-shaped Au/Ag nanorods (NRs) capable of inducing immunogenic cell death (ICD) in tumor cells upon irradiation with 1064 nm light. The corn-like Au/Ag NRs, when combined with NIR-II light irradiation, demonstrated a significant increase in T-cell ICD. Moreover, NIR-II light irradiation led to a notable enhancement in tumor infiltration by T cells, thereby initiating a systemic immune response aimed at reprogramming the immunosuppressed cold tumor microenvironment. These NRs exhibited synergy with the immune checkpoint blockade (ICB) antibodies, effectively inhibiting distal tumor growth and inducing a robust immune memory effect to forestall tumor recurrence [72]. Bai et al. synthesized Ag@CuS-TPP@HA nanoparticles, leveraging the collective properties of reactive oxygen species (ROS), photothermal effects, and ICD antibodies to elicit a potent ICD effect. This, in turn, facilitated dendritic cell (DC) maturation and activation of T-lymphocytes. The combination of ROS, photothermal effect, and ICD antibody stimulation promoted effective ICD, fostering DC maturation, T-lymphocyte activation, and proliferation. Consequently, this approach converted “cold” tumors characterized by low levels of tumor-infiltrating lymphocytes (TILs) into “hot” tumors, bolstering the systemic antitumor immune response. Ultimately, this strategy led to the eradication of primary tumors and suppression of distal tumor growth. The implications of this method extend to providing novel insights for the precise diagnosis of deep-seated tumors and facilitating efficient immune checkpoint blockade (ICB)-based antitumor immunotherapy [73].

#### 3.2.3. Other Combination Therapies

While photothermal therapy (PTT) holds considerable promise for biomedical applications, it also presents certain drawbacks. Apart from the inherent complexity associated with the design of therapeutic apparatus utilizing laser-mediated therapy, several challenges need to be addressed. These include the need for enhanced targeting of light/thermal agents to tumor tissues, the potential accumulation of residual thermal sensitizers, and the risk of collateral damage stemming from stray light, all of which have hindered the widespread clinical adoption of PTT. Moreover, a critical limitation lies in the restricted penetration of light through biological tissues, resulting in diminished efficacy, particularly against deep-seated tumors [19].

**Figure 7:**
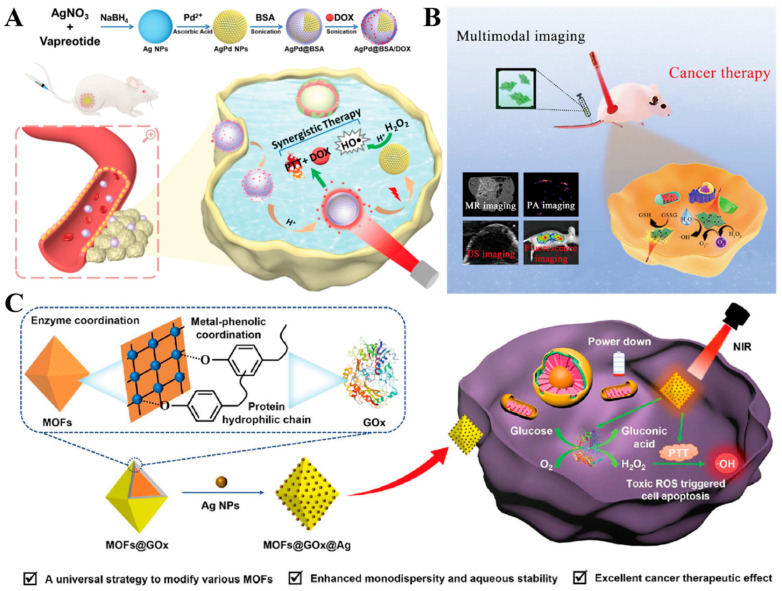
(**A**) Schematic illustration of synthesizing AgPd@BSA/DOX and antitumor mechanism [76]. Copyright 2020, Elsevier B.V. (**B**) Schematic illustration of MoO_3−x_-Ag-PEG-MnO_2_ preparation process and therapeutic mechanism [77]. Copyright 2023, Elsevier Inc. (**C**) Schematic illustration for Enzyme coordination interactions between GOx and MOFs, the modification of AgNPs, and the antitumor mechanism [78]. Copyright 2021, Elsevier B.V.

Based on the inherent limitations of PTT, relying solely on this approach for tumor therapy proves challenging. However, emerging research suggests that integrating PTT with complementary therapeutic modalities often yields synergistic effects, surpassing the efficacy of individual therapies [74,75]. Li et al. devised a strategy involving Ag/Pd bimetallic nanoenzymes with peroxidase-like activity, serving as nanocarriers for adriamycin (DOX). This approach capitalizes on the photothermal conversion capability and catalytic generation of hydroxyl radicals (HO•) to augment antitumor efficacy (Figure 7A). Experimental findings demonstrated that the Ag/Pd nanoenzymes exhibited notably high photothermal conversion efficiency (η = 40.97%) and markedly enhanced peroxidase-like activity upon laser irradiation. Additionally, these AgPdNPs efficiently catalyzed the production of HO• from H_2_O_2_ in an acidic milieu. Upon reaching the acidic tumor microenvironment, the nanomedicine AgPd@BSA/DOX, when subjected to NIR laser irradiation, facilitates DOX release while inducing hyperthermia. This orchestrated approach achieves a multifaceted therapeutic outcome encompassing ROS-mediated tumor ablation, photothermal therapy, and chemotherapy [76]. Gong et al. innovatively engineered Metal–Organic Frameworks (MOFs) by leveraging the intrinsic biogenic enzyme, glucose oxidase (GOx), to attain stable water monodispersity and create active surface sites for subsequent modifications (Figure 7B). Subsequently, silver nanoparticles were uniformly immobilized onto the GOx-functionalized MOFs, enhancing their photothermal conversion efficiency upon exposure to near-infrared light. This integration constituted an efficacious paradigm of combined starvation therapy and photothermal therapy, exhibiting superior efficacy in tumor management and metastasis inhibition compared to singular starvation therapy approaches. Notably, this study introduces a pioneering methodology for enhancing the stability and dispersion of MOFs utilizing bio-enzymes under simplified conditions. Furthermore, it underscores the utility of MOFs in potent tumor therapy strategies, obviating the necessity for conventional chemotherapeutic agents [77]. Wu et al. developed a multifunctional nanoplatform comprising MoO_3−x_ nanosheets, Ag nanocubes, and MnO_2_ nanoparticles. This nanoplatform exhibits dual-mode functionality by generating reactive oxygen species (ROS) and thermotherapeutic effects upon irradiation with 808 nm near-infrared (NIR) light (Figure 7C). Specifically, when MoO_3−x_-Ag-PEG-MnO_2_ accumulates at the tumor site, MnO_2_ effectively depletes glutathione (GSH) with its antioxidant capacity and decomposes hydrogen peroxide (H_2_O_2_) to generate highly cytotoxic hydroxyl radicals (•OH) and oxygen (O_2_), thereby enhancing photodynamic therapy (PDT). The NIR-mediated photothermal therapy (PTT) offers superior tissue penetration compared to visible-light-mediated PDT. Moreover, PDT efficacy can be further enhanced by irradiating MoO_3−x_-Ag, leveraging silver’s strong absorption of NIR light for efficient photothermal conversion. This single nanomaterial integrates NIR laser-induced synergistic PDT/PTT with multimodal imaging capabilities, holding significant promise for cancer diagnosis and therapy [78].

#### 3.2.4. Biological Imaging

Silver-based equipartitioned excitonic nanoparticles find extensive applications in catalytic technology, nanomedicine, and analytical detection, owing to their exceptional optical properties. Therefore, a comprehensive exploration of the optical characteristics of individual silver-based nanoparticles is imperative. When subjected to an optical field, the electrons within these nanoparticles resonate, resulting in the generation of a surface plasmon resonance absorption peak. The position and intensity of this absorption peak are intricately linked to the nanoparticle’s shape, size, dielectric constant, and the refractive index of the surrounding medium.

The optical behavior of metal nanoparticles is significantly influenced by their size. In particular, the shape of the surface excitations or oscillating surface electrons is closely tied to the dimensions of the nanoparticles. For small nanoparticles, where the particle diameters are much smaller than the incident light’s wavelength, the surface-isolated excitons demonstrate uniform polarization along the incident electric field, indicating the presence of the dipole Surface Plasmon Resonance (SPR) mode. Conversely, in the case of large nanoparticles, the surface-isolated excitons exhibit uneven polarization across the nanoparticle with some phase delay. Daedu Lee et al. [79] conducted a comparative analysis of the extinction spectra of silver-containing films (SCF) embedded within a thin polystyrene (PS) layer. The extinction spectra of SCF composed of small-sized silver nanoparticles (SCF1~SCF3 with an average particle size ranging from 59 to 93 nm) predominantly displays dipole SPR bands at wavelengths of between 489 and 557 nm. However, with an increase in particle diameter to 219 nm (SCF8), the dipole SPR band experiences a consistent redshift, extending to 900 nm.

Magnetic resonance imaging [80] (magnetic resonance imaging, MRI) and photoacoustic imaging [81] (photoacoustic imaging, PAI) are currently the preferred medical imaging techniques. Regions of superparamagnetic iron oxides (SPIONPs) aggregation produce strong negative contrast in T2/T2* weighted MR images and appear as dark images with low signals. Furthermore, 16 SPIONPs are easy to aggregate, and their stability is enhanced by a factor of 30 after coating with noble metals, while showing strong negative contrast in MRI and strong NIR (680~850 nm) absorption [63]. Therefore, the development of multifunctional nanoplatforms, consisting of AgNPs and iron oxide nanoparticles (IONPs), can be used to develop MRI and PA imaging modalities. Shehzahdi S. Moonshi et al. [82] designed a novel silver–iron oxide nanohybrid and succeeded in the NIR region with effective targeted photothermal therapeutic strategy and dual imaging capability using MRI (in vitro and in vivo) and PAI (in vitro) for anticancer therapy. The excellent anticancer activity of this nanoparticle system is determined by the inherent anticancer properties of Ag and the elevated photothermal temperature under near-infrared light irradiation. FA–AgIONPs show excellent potential for simultaneous applications in safe and successful targeted photothermal therapy, dual-modal imaging in in vitro MRI, and in vivo imaging of cancer models.

#### 3.2.5. Drug Delivery

The advancement of targeted drug delivery techniques has significantly enhanced the efficacy of nanoparticle-based anticancer therapeutics, particularly those employing metal nanoparticles. In the realm of drug delivery, surface engineering of silver nanoparticles with specific functional groups enables targeted modifications, thereby augmenting their affinity towards the intended target site. Functionalization can involve the attachment of various molecules such as ligands, antibodies, proteins, or oligonucleotides onto the nanoparticle surface. Victoria O. Shipunova et al. conducted pioneering work in synthesizing targeted formulations for cancer photothermal therapy (PTT), which entail combinations of silver nanoparticles (AgNPs) and the anti-HER2 affinity ligand, ZHER2:342. The localized surface plasmon resonance (LSPR) properties of AgNPs are further amplified by heating the targeted nanoparticles within HER2-positive cells, thereby enhancing the therapeutic effect [83]. However, it is imperative to address concerns regarding poor biocompatibility, as it may incite immune responses or toxic effects, thereby compromising the efficacy of targeted delivery and therapeutic outcomes. Thus, ensuring optimal biocompatibility of silver nanoparticles with biological systems emerges as a critical consideration in achieving effective targeting strategies. Renquan Lu et al. conducted an aqueous phase synthesis of silver (Ag) nanoparticles utilizing silver nitrate (AgNO_3_) and freshly extracted egg whites. The proteins present in the egg whites possess diverse functional groups that play pivotal roles in the reduction of Ag^+^ ions and in maintaining the stability and dispersion of the resultant nanoparticles. This process is crucial for achieving the desired properties of the nanoparticles. In vitro cytotoxicity assessments demonstrated that the Ag–protein biocouplings exhibited excellent biocompatibility with the mouse fibroblast cell line 3T3. Furthermore, X-ray irradiation experiments conducted on 231 tumor cells revealed that these biocompatible Ag–protein biocouplings enhanced the efficacy of the irradiation therapy [84].

Through meticulous design and modulation of these variables, researchers can attain a heightened level of targeting specificity with silver nanoparticles, rendering them increasingly auspicious for a spectrum of medical applications. These applications encompass tumor therapy, molecular imaging, and drug delivery, where the unique properties of silver nanoparticles can be harnessed to advance therapeutic and diagnostic modalities.

### 3.3. Platinum Nanoparticles

Platinum nanoparticles exhibit not only commendable photothermal stability but also the potential for synergistic applications with chemotherapy or immunotherapy, thereby demonstrating promising outcomes in cancer therapy platforms. This potential is underscored by the ability of platinum nanoparticles to integrate seamlessly into multifaceted therapeutic approaches against cancer [85]. Zhang et al. [86] have developed a comprehensive therapy system proficient in mediating photothermal therapy (PTT), chemodynamic therapy (CDT), and immunotherapy. Utilizing ultrasonic fragmentation techniques, the particle size of the complex was deliberately reduced, enhancing its migratory capability toward lymph nodes and tumor sites. Notably, yeast microcapsules, owing to their dextran components, prove effective in activating immune responses. They stimulate Dendritic Cell (DC) maturation, induce macrophage polarization, release various cytokines, and activate T cells. The integration of PTT and CDT not only ensures the effective elimination of tumor cells but also elicits an antitumor immune response, thereby extending survival times. In a separate study, Sun et al. [87] synthesized Silicon–Platinum nanocomposites (Si–Pt NCs) through in situ reduction of Pt nanoparticles grown on Silicon Nanowires (SiNW). Leveraging the catalytic activity of Pt NPs and the mesoporous structure of SiNWs, the Si–Pt NCs demonstrated robust Sonodynamic Therapy (SDT) and CDT activities, surpassing the efficacy of pure Pt NPs. The integration of these activities presents a promising avenue for cancer therapy. Furthermore, the mild photothermal effect enhances the combined SDT and CDT therapy substantially.

Lei Zhao et al. [88] described a novel dual mesoporous nanosystem that can be used for photothermal therapy (based on Pt) and in vivo magnetic resonance imaging (based on Gd^3+^ ions) with a simple and mild strategy. They first synthesized mesoporous platinum nanoparticles (mPtNPs) and coated them with mesoporous silica to form mPt @mSiO_2_. Next, they modified the mPt@mSiO_2_ nanomaterials with -NH_2_ to allow for further binding with the Gd DTPA complexes, which ultimately led to the formation of the mPt@mSiO_2_-Gd DTPA nanosystem. The core of this system is the mesoporous structure of mPtNPs, which exhibits excellent photothermal effects under 808 nm near-infrared laser irradiation. In addition, the mesoporous silica with a shell layer formed by the Gd DTPA complex shows potential MR imaging contrast agent applications for both photothermal therapy and in vivo magnetic resonance imaging.

### 3.4. Palladium Nanoparticles

Palladium nanoparticles, as noble metals, possess distinctive attributes including thermal and chemical stability, catalytic activity, and adjustable optical response [89]. Notably, these nanoparticles exhibit a stable photothermal effect characterized by a high photothermal conversion efficiency of 49% and demonstrate significant absorption in the near-infrared spectrum. This property facilitates photothermal–electronic interactions, leading to the generation of heat capable of ablating tumor cells. Prem Singh et al. [90] investigated the synthesis of innovative bimetallic palladium nanocapsules (Pd Ncap), comprising gold bead cores encased within hollow porous palladium shells, and explored their application in the realm of photothermal therapy for cancer therapy. The researchers employed bifunctional carboxy-PEG-thiols as junctions and functionalized Pd Ncaps with the targeting molecule Herceptin to enhance the targeting efficacy against SK-BR-3 cells. The verification of this coupling was conducted through detailed X-ray Photoelectron Spectroscopy (XPS) analysis. The targeted in vitro photothermal therapy (PPTT) efficacy of Herceptin-conjugated Pd Ncap against SK-BR-3 breast cancer cells was assessed, revealing a remarkable cell kill rate of 98.6% at a concentration of 50 μg/mL, utilizing a 1064 nm near-infrared (NIR-II) laser at a low power density of 0.5 W/cm^2^. Xue Dong et al. [91] proposed a multifunctional bioactive gel system designed not only for drug delivery but also to establish a self-adjuvant immune microenvironment that synergistically augments photothermal therapy (PTT), eliciting a potent antitumor immune response. Initially, the M13 phage was engineered via phage display technology to express the glutamate sequence on the pVIII protein (designated as M13E), enhancing biomineralization processes. Subsequently, a self-adjuvant phage gel (referred to as M13 Gel) was synthesized through chemical cross-linking of glutaraldehyde with the engineered phage coat protein, facilitated by a Schiff base reaction. Palladium nanoparticles (PdNPs), possessing photothermal properties, were then mineralized in situ on the surface of the M13 Gel, resulting in the formation of M13@PdGel. Further incorporation of the IDO1 inhibitor NLG919 led to the development of M13@Pd/NLG Gel. This multifunctional bioactive gel system not only facilitates cargo loading but also serves as a self-adjuvant and antigen reservoir, thereby promoting immune cell activation.

Through surface functionalization, palladium nanoparticles can bind to target molecules to enable targeted imaging of specific cells or tissues, leading to potential applications in areas such as cancer diagnosis and treatment monitoring [92]. It is an interesting observation in the literature that bimetallic nanomaterials may exhibit very different synergistic effects than monometallic nanomaterials. This phenomenon can be attributed to the fact that the geometry of the metal particles has an impact on the structure and ratio of the active centers of the catalytic material. Examples include palladium nanorods [93], palladium nanosheets [94], and palladium nanospheres [95]. In addition, due to the quantum size effect, the electronic energy levels of the metal nanoparticles change, which, in turn, affects the orbital hybridization and charge transfer between the catalytic material and the reactants. These structural changes may contribute to the enhancement of enzyme activity and photothermal conversion. Therefore, we can assume that bimetallic nanomaterials have unique properties which are beyond the scope of single-metal nanomaterials. Ruyu Li et al. [96] introduced palladium nanomaterials to improve the catalytic ability and photothermal conversion of silver nanoparticles, and prepared elm pod polysaccharides (EPP-AgPd1.5 NPs) stabilized silver–palladium bimetallic nanoparticles, EPP-AgPd1.5 NPs, which have good photothermal conversion performance and antitumor ability. EPP-AgPd1.5 NPs have good potential for future biologically relevant detection and the therapy of malignant tumors.

## 4. The Advantages and Disadvantages of Noble Metal Nanoparticles

In recent years, noble metal nanoparticles have played a crucial role in the field of biomedical materials. These nanoparticles exhibit remarkable optical properties, particularly localized surface plasmon resonance (LSPR), making them highly desirable for sensor fabrication [97]. The aggregation of noble metal nanoparticles has been shown to significantly enhance their optical characteristics, augment the electromagnetic field, and increase their hydrodynamic diameter. Consequently, sensors based on the aggregation of noble metal nanoparticles demonstrate exceptional performance in detecting harmful substances [98]. Noble metal particles possess outstanding nanophotonic and catalytic properties, along with high surface area-to-volume ratios. Various noble metal nanostructures with diverse sizes, shapes, compositions, and aggregation states have been developed and found widespread applications in catalysis [99], imaging [100], therapeutics [101], and light harvesting [102]. Additionally, noble metal nanoparticles serve as effective nanosensors for detecting and treating cancerous substances. Furthermore, they serve as carriers for drug delivery, facilitating controlled release, and the enhanced and targeted delivery of therapeutic agents, thereby improving therapeutic efficiency for various diseases.

However, noble metal nanomaterials are not without limitations, which can hinder their application in tumor therapy and other scientific domains. The production process of noble metal nanomaterials tends to be more expensive and intricate compared to alternative materials. This inherent complexity can restrict their scalability and elevate production costs, posing challenges for widespread adoption. Additionally, while noble metals generally exhibit stability under standard room temperature and pressure conditions, they are susceptible to oxidation, dissolution, or chemical reactivity under specific environmental circumstances, such as elevated temperatures or exposure to strong acids or bases. These factors can compromise their stability and performance over time [103]. Moreover, certain forms of noble metals may exhibit biotoxicity [79]. For instance, silver nanoparticles (AgNPs) have been found to interact with the intestinal immune system, triggering immune-related signaling pathways that modulate pro-inflammatory and/or anti-inflammatory cytokines, ultimately leading to inflammatory effects [104]. Consequently, despite the numerous advantages offered by noble metal nanomaterials across various applications, these aforementioned drawbacks impose undeniable limitations, particularly in medical fields.

## 5. Conclusions and Future Prospects

In summary, noble metal nanomaterials play a crucial role in biomedical applications and significantly contribute to the advancement of the pharmaceutical industry. Gold nanoparticles, silver nanoparticles, platinum nanoparticles, and palladium nanoparticles are extensively utilized in biomedical research and clinical practice, owing to their remarkable properties. Additionally, noble metal nanoparticles exhibit a potent surface plasmon resonance effect, enabling efficient absorption of specific light wavelengths, particularly in the near-infrared (NIR) region. This phenomenon facilitates the conversion of light energy into heat energy, enabling targeted tumor ablation while minimizing damage to surrounding healthy tissues.

However, the single use of noble metal nanomaterials for drug delivery can have the disadvantage of insufficient targeting, which, in turn, reduces drug delivery efficiency. Therefore, targeted drug delivery can be achieved by careful design and customization of noble metal nanomaterials, along with enhancement of their photothermal properties and stability. Undoubtedly, through strategic modifications, the efficacy of delivering noble metal nanoparticles to specific sites is significantly amplified. Additionally, it is recognized that a singular photothermal therapy (PTT) approach may not always yield the optimal outcome in tumor therapy. Consequently, integrating noble metal nanoparticle-mediated photothermal therapy with immunotherapy, chemotherapy, radiotherapy, or radiation holds promise for synergistic effects, surpassing the additive efficacy principle (“1 + 1 > 2”). Moreover, the precise control over size, shape, and surface properties during synthesis confers versatility upon noble metal nanoparticles, enabling the design and fabrication of tailored nanomaterials for distinct cancer types. Furthermore, owing to their remarkable optical characteristics and enduring photothermal stability, noble metal nanoparticles emerge as compelling contenders for clinical translation, playing pivotal roles in bioimaging, drug screening, and various other biomedical applications. In recent years, although photothermal therapy has made great strides in clinical research, it still faces a number of challenges. The distribution and transportation of most phototherapeutic molecules in organisms is not ideal due to the lack of effective PTAs in clinical practice. However, the limited depth of light penetration restricts the therapeutic effect on large and deep tumors. Therefore, precious metals with high photothermal conversion efficiency are expected to be ideal photosensitizers. However, in recent years, mild PTT has gradually become a research trend. Fortunately, the relationship between molecular structure and phototherapeutic effect is simple and controllable. We believe this will facilitate the rapid development of next-generation phototherapy in clinical applications.

However, noble metal nanoparticles are currently in the early stages of research, and further exploration of their potential is warranted. Particularly concerning their potential toxicity, chemical stability, and synthesis methods, significant challenges persist. In conclusion, while noble metal nanomaterials hold significant promise for photothermal therapy (PTT) applications, ongoing research efforts are essential to fully understand and address their limitations. We must persist in our exploration of their functionalities and strive to overcome current challenges to pave the way for their clinical utilization in combating cancer.

## Figures and Tables

**Table 1:** Summary of the properties and applications of noble metals in tumor photothermal therapy.

Types	Properties	Shapes	Combination Therapy	Biological Imaging	Drug Delivery
Gold nanoparticles	LSPR/stability/ catalytic activity	Gold nanorods/ gold nanocage/ gold nanostars/ gold nanospheres	PTT/ IMT/chemotherapy/PDT	Raman spectroscopy	peptide modification/ Localized heating
Silver nanoparticles	LSPR/stability/ catalytic activity	quasi-spherical silver nanoparticles/ silver nanorods/ silver nanocubes/ Ag-Rh core-framework nanocubes/ silver prismatic nanocubes	PTT/ IMT/bimetallic/ chemotherapy	MRI/PAI	targeted modification/ functionalized
Platinum nanoparticles	LSPR/stability	Platinum Nanorods/ Platinum Nanosheets/ platinum nanospheres	SDT/CDT/ IMT	MRI	functionalized
Palladium nanoparticles	LSPR/stability/ catalytic activity/ adjustable optical response	Palladium Nanorods /Palladium Nanosheets/ Palladium nanospheres	bimetallic/ bioactive gel system/ IMT	MRI	functionalized

## Data Availability

Not applicable.

## Abbreviations

•OH	hydroxyl radicals
PTT	Photothermal therapy
PTA	Photothermal transducer
PDT	Photodynamic therapy
SDT	Sonodynamic therapy
SPR	Surface plasmon resonance
LSPR	Localized surface plasmon resonance
PNPs	Plasma nanoparticles
AuNSs	Gold nanospheres
AuNRs	Gold nanorods
AuNSTs	Gold nanostars
DCs	Dendritic cells
ACT	Adoptive cell therapy
NCs	nanocomplexes
NIPAM	(HPMA)-co-N-(1-vinyl-2-pyrrolidone)
ROS	Reactive oxygen species
THPP	Tetra(4-hydroxyphenyl)porphyrin
NIRF	Near-infrared fluorescence
PAI	Photoacoustic imaging
PA	Photoacoustic
NCT	Nanocatalytic therapy
CQDs	Colloidal quantum dots
PD-1	Programmed death receptor
PD-L1	Programmed death ligand 1
CTLA-4	Cytotoxic t-lymphocyte antigen 4
ICD	Immunogenic cell death
SCF	Sliver-containing films
MRI	Magnetic resonance imaging
CDT	Chemodynamic
DC	Dendritic cell
SDT	Sonodynamic therapy
XPS	X-ray photoelectron spectroscopy
MOFs	Metal–organic Frameworks
OCT	Octreotide
SERS	Surface-Enhanced Raman spectroscopy
H_2_O_2_	hydrogen peroxide
